# Emotion Recognition and Aging. Comparing a Labeling Task With a Categorization Task Using Facial Representations

**DOI:** 10.3389/fpsyg.2020.00139

**Published:** 2020-02-07

**Authors:** Mandy Visser

**Affiliations:** ^1^Improving Palliative, Aged and Chronic Care Through Clinical Research and Translation (IMPACCT), University of Technology Sydney, Sydney, NSW, Australia; ^2^MARCS Institute for Brain, Behaviour and Development, Western Sydney University, Sydney, NSW, Australia

**Keywords:** emotion recognition, categorization, labeling, aging, facial expression of emotion

## Abstract

Research suggests that aging comes with a decline in the ability to identify emotional expressions. In previous studies on emotion recognition and aging, participants were typically instructed to classify images of facial expressions using sets of lexical emotion labels. Yet, in daily life, when exposed to facial expressions by others, people match these with their conceptual knowledge of how emotions are visually presented (i.e., a smile for “happiness”), rather than recalling lexical labels (i.e., the word “happy”). By comparing performances of young adults and older adults on an emotion sorting task based on visual categorization and a traditional labeling task based on lexical categorization, this research aimed to explore a different way of studying emotion recognition abilities over the lifespan. In line with earlier research, results of the labeling task showed that our older participants (*M_*age*_* = 71.9) were less accurate in labeling emotions than participants in a young age group (*M*_*age*_ = 23.8), especially for expressions of sadness, fear, anger and contempt. Outcomes of the categorization task suggest that older adults have difficulties separating distinctive meanings of emotions more than young adults do. Results of this study indeed shows a decline in emotion recognition using both tasks, and suggests future studies to examine possible changes in conceptual knowledge of emotions, rather than the inability to perceive certain facial cues.

## Background

As we age, we seem to have more difficulties with recognizing emotional expressions with others (Calder et al., 2003; Mather and Carstensen, 2003; Ruffman et al., 2008; Krendl and Ambady, 2010; Isaacowitz and Stanley, 2011; Ruffman, 2011; Schlegel et al., 2014). This paper examines differences in emotion recognition by young and older adults, using two different experimental tasks. Traditionally, labeling tasks are used to examine differences between age groups in recognizing and labeling emotions from facial expressions (Calder et al., 2003; Mather and Carstensen, 2003; Ruffman et al., 2008; Isaacowitz and Stanley, 2011; Ruffman, 2011; Schlegel et al., 2014). In these tasks, participants are typically instructed to classify visual representations (e.g., a picture or a video clip of a smiling face) by choosing a lexical label (e.g., the word “happy”). However, in daily life, we tend to recognize emotional expressions by comparing them with existing mental representations of emotions (categorizing) rather than naming these expressions (labeling) (Feldman Barrett, 2006). Hence, using categorization tasks instead of labeling tasks to test emotion recognition abilities may be more ecologically valid, as this would represent real life processes to a larger extend than lexical labeling. Using labeling tasks may affect participants’ performance for the wrong reasons, as retrieving specific names of emotions from one’s memory is cognitively demanding (Barber and Mather, 2013). This paper describes performances of young and older adults on a typical labeling task and a categorization task. In this way, this research aims to contribute to a better understanding of how emotion recognition abilities change over the lifespan, and explores appropriate methodologies.

The ability to recognize emotions by interpreting another person’s non-verbal expression (in the face, voice or body) is essential for effective communication (e.g., Ekman and Oster, 1979; Knapp and Hall, 2010). It is an important precondition for understanding and reacting appropriately to someone’s behavior and therefore crucial for partaking in social interaction (Schlegel et al., 2014). The meaning of a message is often affected by a person’s characteristics, such as the (emotional) features of the sender’s face (Knapp and Hall, 2010). For example, the assumed underlying intention of a question like, “madam, can you please move your chair,” can be deduced from the emotional signals someone is cueing with this message. One can smile, and signal happiness, or one can press his or her lips, showing anger. Not comprehending these facial cues and recognizing them as signs of specific emotions being present would lead to a lack of understanding of what another person is feeling and intending to convey. This affects the ability to communicate effectively. A lower sensitivity to others’ emotional expressions is associated with antisocial behavior (Marsh and Blair, 2008) and the ability to recognize emotions accurately is related to better social skills and a higher quality of social relationships (Hall et al., 2009).

Overall, studies indicate that emotion recognition changes over the lifespan (for meta-analyses, see Murphy and Isaacowitz, 2008; Ruffman et al., 2008; Reed et al., 2014). Depending on the specific emotion (e.g., happiness, sadness) and the modality in which it is presented (e.g., visual, audio), older adults are generally less accurate recognizing emotions, compared to young adults (Calder et al., 2003; Mather and Carstensen, 2003; Ruffman et al., 2008; Krendl and Ambady, 2010; Isaacowitz and Stanley, 2011; Ruffman, 2011; Schlegel et al., 2014). It has been argued that this decline would be valence-related: older adults are supposed to be worse in recognizing negative emotions, like anger and fear, than in positive emotions, like happiness (Mather and Carstensen, 2003).

Earlier studies attempt to explain this decline in recognition of negative emotions in various ways. First, older adults are presumed to primarily focus on the lower half of faces (e.g., mouth region) when communicating with others, as shown in emotion recognition studies using eye-tracking methods e.g., (Sullivan et al., 2007). Therefore, they may fail to recognize emotions that are expressed mostly in upper parts of the face. This would explain why older adults are worse at recognizing anger and fear, emotions that are mainly expressed in the eye region, as compared to happiness that is arguably expressed by the use of smiles. A second possible reason for a decline in the ability to recognize negative emotions with age is the proposed positivity bias in older adults (Carstensen et al., 2006). More specifically, older adults tend to try maximizing emotional rewards in the context of social interaction and concentrate on positive social interaction, rather than negative interaction. Therefore, with aging, people tend to experience fewer negative emotions and are argued to be worse in recognizing and distinguishing negative emotions (Mather and Carstensen, 2003).

However, these arguments seem unlikely to account for all decline in emotion recognition. For example, emotional expressions of anger and sadness, both defined as negative emotions, are not limited to upper face regions and expressed in the lower half of the face as well (Mill et al., 2009). Additionally, mixed findings are shown when studying the recognition of specific emotions (Isaacowitz and Stanley, 2011), contradicting the positivity bias theory by Carstensen et al. (2006). For example, meta-analytic studies by Murphy and Isaacowitz (2008) found that, next to happiness, the (negative) emotional expression of sadness is also accurately recognized by older adults, opposed to meta-analyses by Ruffman et al. (2008), who do claim that sadness recognition was impaired by older adults. Mill et al. (2009) did not find a shift toward positive emotions in confusion matrices at all. Apparently, it is still unclear what mechanisms underlie the decline of emotion recognition in aging.

The current paper takes a different approach to clarify effects of aging on emotion recognition, exploring the possibility that the methodology used in emotion recognition research may account for these conflicting arguments. In daily life, we use categorization schemes to give meaning to facial expressions, we do not necessarily give them a lexical label. For example, when in conversation with someone, who frowns and presses his or her lips together, we know how to interpret these features without the necessity to name the emotion that these features belong to (in this case, anger). This is because we use categorization strategies to give meaning to emotions. According to Feldman Barrett (2006), emotion recognition with others only occurs when the emotional expression of that person is categorized. Categorization is the cognitive process of grouping things that have more common attributes and separating those that have fewer common attributes and is crucial to the processes of recognition (Gati and Tversky, 1984; Tversky and Hemenway, 1984; Canter et al., 1985). Meanings of emotional expressions are shaped by concepts of emotion categories we have in our mind. Hence, it may be more informative to study the effect of aging on the way we categorize or group emotions, rather than the effect of aging on the ability to name certain typical expressions.

## The Current Study

This research explores differences between young and older adults on their performance on recognizing facial expressions of emotions in a (semi-open) emotion categorization task, and compares any differences with their performance on an emotion-sorting task using lexical labels.

Earlier studies using typical sorting tasks show differences in emotion recognition between young adults and older adults. Therefore, we expect older adults to be worse in their overall performance in the sorting task using lexical labels, compared to young adults. Moreover, based on earlier studies, we expect older adults to perform worse on negative emotion recognition, compared to positive emotion recognition. In the (semi-open) categorization task, we ask participants to form groups of existing images showing emotions. If older adults have more difficulties with categorizing emotions than young adults, they are expected to create more heterogeneous groups than young adults. That is, their groups of emotions are expected to contain more emotions than the groups of young adults, as they would make more mistakes in forming the groups. If older adults are as good in recognizing emotions as young adults, and their failing in labeling task is because of the nature and level of difficulty, a difference between the age groups is expected for the labeling task, similar to earlier studies, but not necessarily for the categorization task.

## Method

### Participants

A total of twenty young adults (10 female), with a mean age of 23.80 years (*SD* = 4.27), and twenty older adults (11 female), with a mean age of 71.85 years (*SD* = 6.93), participated in this study. All young adults were undergraduate students of Western Sydney University, Australia, and partook in the experiment voluntarily. Senior participants were recruited at communal computer clubs for older adults located in different suburbs of Sydney, Australia, and were compensated accordingly for their efforts to travel to the campus of Western Sydney University. Prior to the experiment, participants were subjected to an extensive screening, including mental health assessments, cognitive tests, and sight and hearing examinations. We ensured all participants were cognitively and emotionally healthy at the time of participation, and no one showed any signals of substantial hearing and/or sight deficiencies. Beforehand, participants signed a consent form by which they gave permission their data to be used for scientific purposes. Approval for the study was obtained from the ethical committee of Western Sydney University; recruitment and experimental procedure followed the guidelines of the Institutional Review Board.

### Stimuli

Participants were asked to categorize emotional expressions in two different tasks. For these tasks, we used two paper card decks that both contained 64 emotion expression cards. The digital color images that were used as emotional expression cards were selected from the Radboud Faces Database (RAFD, Langner et al., 2010), and showed typical facial expressions for eight defined emotions (anger, disgust, fear, happiness, sadness, surprise, contempt, and neutral). For the RAFD, 49 models were trained to show a range of typical emotional expressions according to the Facial Action Coding System (FACS, Ekman and Friesen, 1978). FACS is an objective system that decomposes facial behavior into components, i.e., Action Units. For example, happiness is characterized as the Action Union pattern of cheek raiser (AU 6) and lip corner puller (AU12). For this experiment, we chose images of four female and four male models in the RAFD, based on validation scores (all above an average of 86 percent). We used both frontal as left side profile images for our card decks. All cards were semi-randomly distributed in such a way both card decks contained all eight emotional categories represented by all eight models, either frontal or left sided.

### Procedure

The experiment consisted of two parts, in which participants first had to complete a semi-open categorization task, followed by a labeling task, using the two decks of emotional expression cards. All participants completed the labeling task after the semi-open categorization task, as this prevented participants to use labels provided in the labeling tasks as suggestions of possible categories in the semi-open categorization task. Participants were instructed to sit at a table, large enough to spread out all 64 cards, in front of the experimenter. The experimenter told the participants they were going to play two card games, using cards showing faces.

For the semi-open categorization task, participants were instructed to sort the cards into eight groups that represented eight emotional categories. It was not specified what emotions were displayed on the cards, into which categories the participant had to group them, or how many cards should one group contain. Participants were told they were free to rearrange, break or reassess the groups until they found an arrangement of eight groups they were content with. The semi open nature of this task, the instruction to sort the cards into a fixed number (eight) of groups, was chosen as pilot work showed both young and senior participants were uncomfortable with the difficulty of a completely open task. Telling participants how many groups actually presented different emotions helped them in completing the task in a reasonable time. Although participants were urged to complete the task as fast as possible, there was no time constraint.

Next, in the labeling task, eight lexical labels were laid out on the table in front of the participant, each label describing an emotional expression (sadness, happiness, fear, anger, disgust, surprise, contempt, and neutral). When handed out the second card deck, participants were asked to lay the cards, one by one, under one of the labels with the image facing down. They were not allowed to reconsider their choice. In this way, participants were forced to compare the cards with the lexical labels, and prevented them to compare them with other emotion expression cards. Similar to the categorization task, participants were urged to complete the task as quickly as possible.

## Results

### Semi-Open Categorization Task

In the semi-open categorization task, participants were given a deck of 64 emotional expression cards and instructed to create eight groups, based on the emotions the faces on the cards expressed.

On average, it took young adults 304 s (*SD* = 110 s) to sort all cards into eight categories. Senior participants needed 536 s (*SD* = 182 s) to complete the semi-open categorization task. Young adults sorted the cards in groups varying in size from 2 to15 cards per group (*SD* = 1.73). Older adults’ group sizes varied from 2 to 21 cards per group (*SD* = 2.66). Focusing on what emotional expressions participants categorized together, repeated measure analysis of variance showed that young adults grouped a smaller amount of different emotions together (*M_*young adults*_* = 1.49 emotions in one group, *SD* = 0.76) than older adults did (*M*_*older adults*_ = 2.09 emotions in one group, *SD* = 1.17), *F*(1,37) = 11.15, *P* = 0.002. This shows that older adults made more “mistakes” than young adults when categorizing the emotional expressions, considering the labels that the expression cards were given by the RAFD database (2010).

This categorization task is a form of exclusive centroid clustering, meaning data points (an x amount of cards of the 64 emotional expression cards per participant), were exclusive to one cluster (1 of the 8 emotion categories), and clusters were predefined (as an emotion). In order to determine differences between young and older adults for how they clustered the 64 emotional expressions, we counted how many times an emotion card was matched with a specific other card, and calculated the percentage of which the particular emotion was clustered with other emotions. Using Pearson’s chi-square, we found significant differences between age groups comparing differences between the main emotion clusters (e.g., fear) and the other emotions (e.g., non-fear, all emotions other than fear together). Table 1 reports on Pearson’s chi-square tests for all emotions, and gives a descriptive overview of found clusters (in percentages). For example, focusing on how participants grouped typical happiness expressions, young adults grouped typical happiness expressions in 98.21 percent of the categorizations with typical happiness expressions. 1.43 percent of all typical happiness expression cards were grouped with typical neutral expressions and 0.36 percent was grouped with typical anger expressions. Older adults grouped typical happiness expressions in 83.36 percent of the categorizations with typical happiness expressions, 11.38 percent of all typical happiness expression cards were grouped with typical neutral expressions, 1.19 percent with contemptuous expressions, 0.85 percent with anger, 0.17 percent with fear, and 3.56 percent with surprise. For all other emotion clusters, please see Table 1.

**TABLE 1:** Emotion clusters in the sorting task (in percentages) by young adults and older adults and Pearson’s chi-square scores representing differences between age groups for frequencies of main emotion clusters (in bold) compared to clusters with other emotions.

	*Happy*	*Neutral*	*Cont.*	*Disgust*	*Anger*	*Sad*	*Fear*	*Surprise*
**Young adults**								
Happy	**98.21**	1.12	0	0	0.30	0	0	0
Neutral	1.43	**78.33**	12.81	0	3.18	1.97	0	1.12
Contemptuous	0	14.20	**60.24**	4.64	16.32	4.28	2.04	1.69
Disgust	0	0	4.36	**64.70**	17.29	0.33	7.07	5.50
Anger	0.36	3.37	15.60	17.58	**49.79**	13.98	1.22	0.14
Sad	0	1.76	3.43	0	11.76	**73.68**	4.08	0.71
Fear	0	0	1.98	7.31	1.24	4.93	**61.63**	24.82
Surprise	0	1.12	1.59	5.49	0.14	0.82	23.95	**66.01**
Total	100	100	100	100	100	100	100	100
**Older adults**								
Happy	**83.36**	8.50	0.67	0	0.50	0	0.10	2.10
Neutral	11.38	**56.98**	14.94	1.70	4.80	4.05	0.83	0.81
Contemptuous	1.19	19.80	**31.23**	17.69	17.02	13.72	4.79	4.77
Disgust	0	2.16	16.86	**37.49**	14.91	10.69	12.80	7.21
Anger	0.85	6.09	16.28	14.97	**30.73**	24.30	8.74	1.74
Sad	0	4.57	11.69	9.55	21.62	**39.60**	5.52	1.74
Fear	0.17	1.02	4.41	12.36	8.41	5.96	**31.22**	40.23
Surprise	3.56	0.88	3.93	6.23	0.20	1.69	36	**40.81**
Total	100	100	100	100	100	100	100	100
**Pearson’s χ**	74.05*	75.32*	150.34*	122.88*	64.22*	168.63*	156.07*	98.86*

*P < 0.001.

### Labeling Task

In the labeling task, participants were given a second deck of 64 emotional expression cards and were instructed to place them one by one under one of the eight lexical labels presented.

**FIGURE 1:**
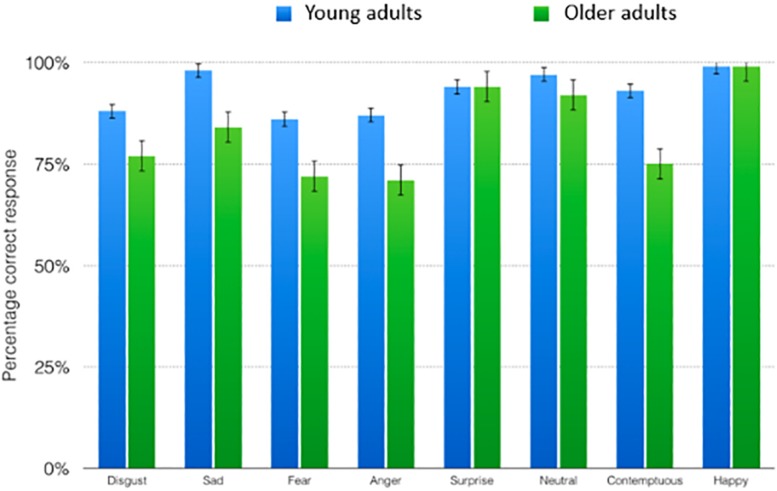
The percentage correct responses for all emotion labels in the function of age group.

Young adult participants needed 242 s (*SD* = 47 s) to complete the labeling task. Senior participants took 340 s on average (*SD* = 117 s) to name all 64 emotional expressions in the card deck. A repeated measures analysis of variance with age group as a between factor and emotion as within factor showed a main effect of age group on emotion recognition accuracy (percentage correct responses), *F*(1,39) = 16.88, *P* < 0.001 η^2^ = 0.31 In general, young adults were more accurate than older adults, when labeling the emotions presented to them (young adults *M* = 0.93, *SD* = 0.11, older adults *M* = 0.83, *SD* = 0.11). We also found a main effect of emotion, *F*(1,39) = 10.23, *P* < 0.001 η^2^ = 0.21, and an interaction between emotion and age group, *F*(1,39) = 2.3 *P* = 0.03 η^2^ = 0.06. *Post hoc* simple effect analyses show that older adults are less accurate than young adults when labeling typical expressions of sadness, fear, anger and contempt. Figure 1 shows a graphical representation of the correct responses for all emotion labels in the function of age group (in percentages) and Table 2 shows an overview of simple effect analyses with percentage correct responses in the function of age group.

**TABLE 2:** Overview ANOVA’s with percentage correct responses in the function of age group.

	Young adults		Older adults					
	*M*	*SD*	*M*	*SD*	*F*	*df*	*p*	ηp*^2^*
Happy	0.99	0.03	0.99	0.03	0.00	39	1.000	0.00
Neutral	0.97	0.07	0.92	0.15	1.88	39	0.178	0.05
Contemptuous	0.93	0.10	0.75	0.29	6.95	39	0.012	0.16
Disgust	0.88	0.12	0.78	0.22	2.74	39	0.106	0.07
Anger	0.88	0.20	0.71	0.22	6.38	39	0.016	0.14
Sad	0.98	0.05	0.84	0.16	14.46	39	0.001	0.28
Fear	0.86	0.16	0.72	0.25	4.85	39	0.034	0.11
Surprise	0.94	0.06	0.94	0.10	0.00	39	1.000	0.00

**FIGURE 2:**
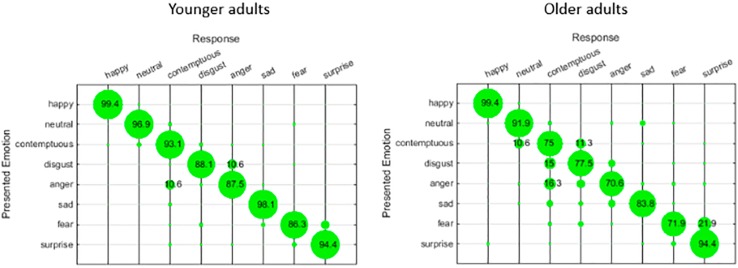
Confusion matrices for presented emotions and responses by young adults and older adults (only percentages >10 are displayed).

So, the results of our labeling task show that older adults performed worse in recognizing sadness, fear, anger and contemptuous expressions than young adults. The confusion matrices in Figure 2 show responses of young adults and older adults when presented with an emotion. Young adults confuse sadness mainly with contemptuous, whereas older adults confuse sadness with contemptuous, disgust and anger. Fear is confused by young adults with surprise, as do older adults. Young adults confuse expressions of anger with expressions of contemptuous, older adults confuse anger with contemptuous and disgust. Finally, contemptuous expressions are mistakenly judged as neutral and disgust by both young adults and older adults.

## Discussion

The aim of this research was to examine the ability to recognize emotions in facial expressions for young and older adults, using a semi-open categorization task and a labeling task.

In line with earlier research, we found that older adults were worse in their overall performance in the sorting task using lexical labels, compared to young adults. As expected, we found that older adults performed significantly worse than young adults, in recognizing expressions of sadness, fear, anger and contempt.

We reasoned that if it was the case that older adults have more difficulties with categorizing facial expressions of emotions than young adults, they were expected to create more heterogeneous groups than young adults. This was indeed what we found. The category groups of emotions older adults formed contained more originally distinct emotions than the groups formed by young adults. Moreover, the size of the formed emotion categories based on facial expressions were larger for older adults than for young adults. Older adults sometimes grouped 21 cards out of the card deck as one category, meaning that they selected one third of the card deck for one out of the eight categories to be formed. This indicates that difficulties experienced by older adults doing emotion recognition tasks in facial expressions are not solely due to having to map facial features onto labels; they also have issues recognizing facial features of emotions.

We expected that a possible decline of emotion recognition to be due to methodology issues that come with labeling tasks, and older adults would not have more difficulties with the categorization task than young adults. This was not the case. Overall, the semi-open categorization task seemed relatively more difficult for older adults. Focusing on the four emotional expressions that were hardest for older adults to recognize in the labeling task (anger, sadness, fear, and contempt), we found in the semi-open categorization task similar large differences between young adults and older adults for all emotional expressions, including these four. Older adults seemed to have difficulties separating facial features. For example, while doing the semi-open categorization task, a number of senior participants thought they were tricked by the experimenter as they found cards showing fear and surprise expressions identical.

Focusing on which specific emotional expressions got confused by participants, or were grouped as one emotion category, we find that those were not related based on facial features. For example, anger and contempt are very distinctively expressed, by different typical facial action units, so does sadness compared to disgust. However, anger and contempt, and sadness and disgust were often confused or grouped as one specific emotion by senior participants. This can be explained in different ways. We suggest an explanation can be sought in similar action tendencies of such emotions and future research should focus on categorization by the meaning of an emotion, rather than by its facial features. This suggestion is line with research by Feldman Barrett (2006) who states emotions are social constructs, and the perception of emotions should be treated more as an assessment of a social situation than as an observation of features. We suggest that a decline in emotion recognition may possibly be due to different interpretation of an emotion, and not necessarily because the inability to perceive certain facial cues. However, as participants were not inquired on their choices in a structural manner no assumption can be made. Contextual factors and interpretations of emotions other than by facial features are important topics to study emotion recognition with older adults, or any participant group for that matter.

This study explored performances of young adults and older adults, using two different emotion recognition tasks with young adult emotional expression cards. Earlier studies on face recognition, using different paradigms and stimuli-modalities, showed inconsistencies around any in-group advantages related to age, possibly affecting performance of older adults when exposed to younger faces stimuli (Anastasi and Rhodes, 2005; Wright et al., 2008; Ebner et al., 2013; Fölster et al., 2014). In general, in-group advantages occur when participants are surrounded by an (in-)group, being exposed to these faces but also sharing values and social rules, opposed to the other (out-)group. For example, in-group advantages for participants of western background opposed to participants of Asian background have appeared in research using western faces stimuli. However, in the case of older adults, values and social rules are still presumably shared with young adults, and older adults are likely to be exposed to the presence of different age groups throughout their life. However, the use of a range of younger and older faces stimuli would be desirable in future work, as it would be a more suited representation of day-to-day life.

## Conclusion

In conclusion, this research contributes to the idea that the methodology used for examining emotion recognition in facial expressions may affect outcomes. This study explored differences between labeling and semi-open categorization paradigms. The attribution to found differences remains unclear and may urge researchers to take a different approach when studying emotion recognition, taking into account the effect of context on the interpretation of facial expressions and emotions, moving toward paradigms that come closer to representing real life emotion recognition.

## Data Availability Statement

The datasets generated for this study are available on request to the corresponding author.

## Ethics Statement

The studies involving human participants were reviewed and approved by the Western Sydney University. The patients/participants provided their written informed consent to participate in this study.

## Author Contributions

The author confirms being the sole contributor of this work and has approved it for publication.

## Conflict of Interest

The authors declare that the research was conducted in the absence of any commercial or financial relationships that could be construed as a potential conflict of interest.
